# Photobiomodulation with Blue Light on Wound Healing: A Scoping Review

**DOI:** 10.3390/life13020575

**Published:** 2023-02-18

**Authors:** Thais P. Prado, Flávia Cristina Zanchetta, Beatriz Barbieri, Caroline Aparecido, Maria Helena Melo Lima, Eliana P. Araujo

**Affiliations:** 1 School of Nursing, University of Campinas (Unicamp), Campinas 13083-887, Brazil; 2 Laboratory of Cell Signaling, Obesity and Comorbidities Research Center, University of Campinas, Campinas 13083-864, Brazil

**Keywords:** photobiomodulation therapy, blue light, low-level light therapy, blue laser, wound healing

## Abstract

Background: Photobiomodulation consists of inducing healing by irradiating light. This scoping review investigates the effect of blue light on the healing process. Methods: The MEDLINE, Web of Science, Scopus, and CINAHL databases were searched. Two reviewers independently examined the search results and extracted data from the included studies. A descriptive analysis was performed. Results: Twenty-two articles were included. Studies were categorized as in vitro/mixed, preclinical, and clinical. The power density used was 10–680 mW/cm^2^ in most of the in vitro/preclinical studies, the irradiation time ranged from 5 s to 10 min, and different wavelengths and energy densities were used. In clinical studies, the wavelength ranged from 405 to 470 nm, and the energy density varied from 1.5 to 30 J/cm^2^. Conclusions: A low energy density (<20 J/cm^2^) was able to stimulate the different cell types and proteins involved in healing, while a high energy density, 20.6–50 J/cm^2^, significantly reduced cell proliferation, migration, and metabolism. There is a great variety of device parameters among studies, and this makes it difficult to conclude what the best technical specifications are. Thus, further studies should be performed in order to define the appropriate parameters of light to be used.

## 1. Introduction

The healing process is a complex physiological response that involves several cellular and molecular events to restore the integrity and functionality of the injured skin. Didactically, it is divided into four overlapping phases: hemostasis, inflammation, proliferation, and remodeling [1]. Wound healing occurs through the activation of different cells, such as keratinocytes, fibroblasts, endothelial cells, macrophages, and platelets, which, in turn, release growth factors, chemokines, and cytokines, contributing to tissue repair [2]. This sequence of events normally occurs in most acute wounds. However, some intrinsic and extrinsic factors may contribute to delayed tissue repair. Among these factors are age, hypoxia, obesity, diabetes mellitus, use of corticosteroids, the presence of biofilm, and local infection, which can impair wound healing and increase the risk of local and systemic complications [3]. The treatment of skin lesions represents a considerable portion of health costs for society, the patient, and their family, on top of impairing the individual’s quality of life [4].

**Figure 1:**
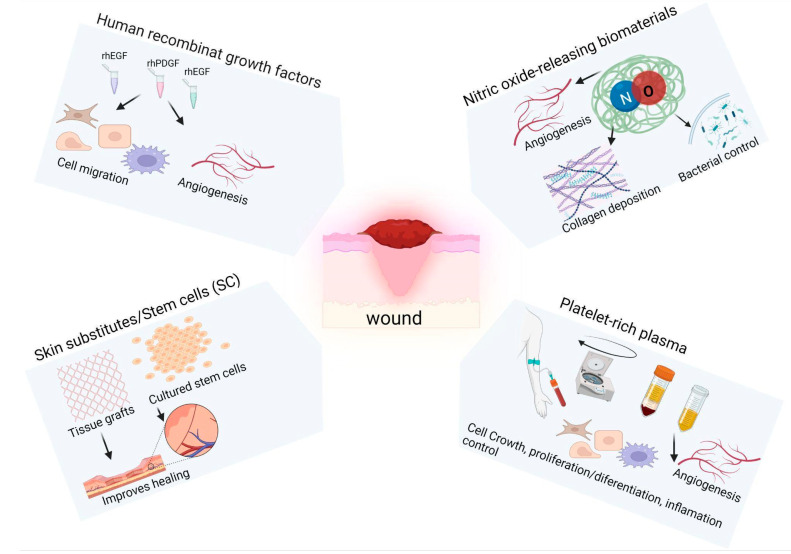
Advanced wound-healing therapies. Wound healing and tissue regeneration can be accelerated by recombinant human growth factors, nitric oxide (NO)-impregnated dressings, tissue grafts made from cultured stem cells, and platelet-rich plasma. Created with BioRender.com (accessed on 11 January 2023).

In the last decade, understanding the molecular and cellular mechanisms of wound healing and tissue regeneration has caused an advance in the expansion of new therapeutic approaches, such as the development of bioengineered products, including recombinant growth factors (GFs), skin substitutes, and platelet therapy, among others (Figure 1) [5]. GFs are essential for skin repair [6]. Topical administration of GFs has been studied over the last few decades and some products have already been placed on the market [7]. However, like other advanced products, GFs alone do not seem to solve all the problems of impaired wound healing.

Among the most studied is recombinant human platelet-derived growth factor (rhPDGF). PDGF is released during hemostasis by activated platelets to promote the migration of neutrophils, macrophages, and fibroblasts, and to induce the formation of granulation tissue and formation of new blood vessels [8]. Application of rhPDGF-ββ hydrogel led to more complete healing of diabetic foot ulcers [9]. Recombinant human epidermal growth factor (rhEGF) is another option that has been well investigated. EGF is produced by activated platelets, M1 and M2 macrophages, and fibroblasts [10]. Topical rhEGF administration led to the complete healing of superficial ulcers in patients with diabetes [11]. Fibroblast growth factors 1 and 2 (FGF1 and FGF2) are produced by most skin cells; they can increase keratinocyte, endothelial cell, and fibroblast migration, promoting reepithelialization and granulation tissue formation [12,13]. Recombinant human fibroblast growth factor 1 and 2 (rhFGF1 and rhFGF2) are effective for skin ulcers and burns, respectively.

Nitric oxide (NO)-releasing biomaterials are another kind of bioengineered products that can improve cutaneous wound healing. NO is produced endogenously and has several physiological functions that are concentration dependent. Therefore, exogenously produced NO-releasing materials can be formulated to have functions such as infection control, collagen deposition, and the formation of new blood vessels [14]. A study that investigated the use of NO nanoparticles (NO-np) demonstrated healing of the injured area in a shorter time compared with the placebo group. NO-np modified leukocyte migration and increased the production of transforming growth factor β (TGF-β) in the wound area, which promoted modulation of angiogenesis and reduced healing time. Using human dermal fibroblasts, NO-np was able to increase fibroblast migration and collagen deposition in injured tissue, demonstrating that NO-releasing nanoparticles accelerate wound healing in a pleiotropic manner [15]. Although the effects related to these approaches for the treatment of skin wounds are positive, the methods used to prepare potential therapies are still limited [16].

Skin substitutes have been another important option for healing wounds that do not respond to conventional treatments. They are based on tissue grafts, cells, or stem cells (SCs) and their derivatives. Autografting is the placement of a thin layer of skin, which includes the entire epidermis and part of the dermis, from a donor area at the wound site. Because this transplant is taken from the patient’s own tissue, there is no risk of rejection [17]. However, when autograft is not possible, an option is allografting, which is based on transplanting tissue from a donor (cadaver or living donor) transplanted into the recipient. The disadvantages of allograft transplantation are that this replacement is temporary due to the possibility of immunogenic host rejection, in addition to the risk of viral transmission [18]. Despite the significant advances that have been made in transplants, the results are not always satisfactory due to failure in the angiogenesis process and the decrease in cell infiltration in the transplanted tissue [5,19].

Recent advances in wound care are the clinical use of autogenous keratinocytes as well as SCs cultured in vitro by bioengineering [5]. SCs have an increased capacity for self-renewal, longevity, and maintenance of physiological functions. SCs can be obtained from the hair follicle, isthmus, infundibulum, and interfollicular epidermis in addition to bone marrow, mesenchymal SCs from adipose tissue, peripheral blood, and umbilical cord/placenta. However, the ideal mechanism for the delivery of SCs to the wound bed and the amount required is not well established in the literature [20].

An option that has been widely used in recent decades is platelet therapy. Platelets play a fundamental role in blood clotting and contain GFs that regulate homeostasis, fibrin clot formation, and tissue repair [21,22]. Platelet concentrate preparations stimulate the regeneration process depending on the number and density of platelets, the type of leukocytes trapped in the fibrin mesh, and the release of active molecules at the sites of injury [22]. The mechanism of action of platelet-rich plasma is release of GFs by activated platelets, such as TGF-β, PDGF, vascular endothelial growth factor (VEGF), and EGF. In turn, these GFs lead to angiogenesis, cell growth, proliferation, and differentiation, in addition to regulation of the inflammatory process [23,24]. Platelet-rich plasma is easy to generate using blood collected from the patient and can be considered a non-invasive treatment. However, the healing mechanism promoted by this therapy has not been fully elucidated. Moreover, blood cannot be drawn from some patients due to hematological problems [5].

The search for new therapies and adjuvant treatments has increased in recent years and has driven new studies with promising results. Furthermore, it is necessary to guarantee a favorable environment for healing, such as adequate wound bed humidity, protection against infectious agents and trauma, cleaning devitalized tissue, and preventing the formation of biofilms [25].

### 1.1. Photobiomodulation and Wound Healing

The use of photobiomodulation (PBM) to treat skin lesions of different etiologies has grown significantly in recent decades, including lesions in the lower limbs of patients with diabetes, pressure lesions, and lesions of venous origin [26]. PBM, also known as a non-thermal treatment, is the use of low-intensity light energy sources for simple tissue healing to reduce pain and inflammation, to accelerate cell repair mechanisms, and to stimulate cell proliferation and the regeneration process [27].

The wavelength of visible light varies from 400 to 700 nm. It can be divided by color: violet/blue (400–500 nm), green (500–565 nm), yellow (565–590 nm), orange (590–625 nm), and red (625–700 nm). Infrared light extends beyond 700 nm. Visible light is distinguished by its wavelength, with different colors reaching different chromophores in the tissues. Melanin, heme, and opsin (OPN) photoreceptors are the main visible light chromophores in the skin [28]. Melanin has an absorption spectrum of 200–900 nm; thus, it is activated by ultraviolet, visible, and infrared light. Hemoglobin has an absorption in the wavelength of blue (418 nm) and yellow/orange (542/577 nm) light, due to the concentration of erythrocytes. Non-image-forming OPNs have wide absorption in different skin cells and regions, such as the epidermis and dermis, including keratinocytes, melanocytes, fibroblasts, and hair follicle cells [28].

**Figure 2:**
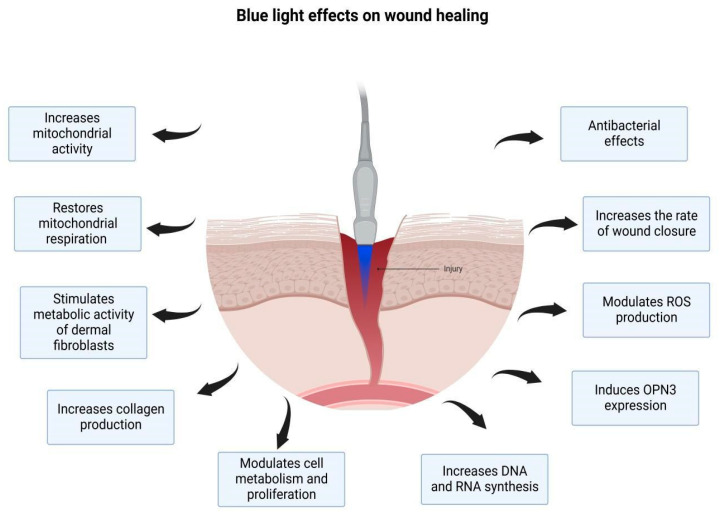
Effects of photobiomodulation (PBM) on wound healing. PBM promotes vasodilation and angiogenesis through the actions of nitric oxide (NO), improving blood supply and stimulating adenosine triphosphate (ATP) synthesis, fibroblast activity, and collagen deposition. Created with BioRender.com (accessed on 11 January 2023).

Studies have shown that PBM offers several benefits in tissue repair: promoting vasodilation and angiogenesis through NO actions; improving capillary and vascular circulation; stimulating synthesis of adenosine triphosphate (ATP), a vital energy source for the metabolism of all cellular processes, including wound healing; stimulating fibroblast activity and collagen deposition; and improving connective tissue repair (Figure 2) [29,30].

In addition, there are recent studies that associated compounds in a nanoparticle system with PBM [31,32]. A study that associated lipid nanoparticles and hyaluronic acid with PBM demonstrated accelerated excisional wound healing, improved resolution of acute inflammation, a reduction in the wound area, a reduction in pro-inflammatory cytokines, an increase in anti-inflammatory cytokines such as a TGF-β, and attenuation of oxidative stress [31]. Another study that used the combination of PBM and curcumin-loaded iron oxide nanoparticles demonstrated significantly improved wound healing in diabetic rats compared with either treatment alone, promoting a significant decrease in macrophages and degranulation of inflammatory cells [32].

There is a lot of interest in the use of PBM in clinical practice, due to its easy and quick application, with less discomfort to the patient, better access to deep wounds, and the possibility of application to large areas. Nevertheless, data have shown that lower doses of visible light are stimulatory, while higher doses are inhibitory [28].

Visible light can also be applied in the form of photodynamic therapy (PDT) [33]. PDT employs a combination of non-toxic dyes (called photosensitizers) combined with visible light for the subsequent formation of reactive oxygen species (ROS), intending to eliminate microorganisms, cancer cells, and unwanted tissues [34]. Although beneficial, the use of PDT has some disadvantages. Patients may report pain and other phototoxic lesions may occur, such as, erythema, edema, and urticaria that tend to disappear in a few days; however, persistent erythema may develop for several months after treatment. In addition, erosions, infection, and sterile pustules are possible adverse effects, although they are rare. In the long term, pigment changes and scarring can occur [33].

### 1.2. Blue Light and Wound Healing

The knowledge about the actions of blue light (BL) on wound healing has been quite controversial [35]. Studies have shown that BL, under certain wavelength ranges and light exposure conditions, can be toxic to several cell types, including keratinocytes, fibroblasts, retinal epithelial cells, and skin-derived endothelial cells [33]. Moreover, the penetration depth of BL is low in the skin tissue (about 1 mm) compared with red light (RL; 4–5 mm) or infrared light (5 mm), a factor that could decrease its effectiveness [36].

On the other hand, studies have demonstrated that the use of the specific frequency band of BL, between 400 and 500 nm, can be used to treat acne vulgaris [37,38], psoriasis [39,40], eczema [41], and skin lesions by decreasing inflammation, reducing the release of pro-inflammatory cytokines [42].

Another important effect described is to reduce bacterial growth in the wound bed [43,44,45]. This effect has stimulated researchers’ interest because the presence of bacteria delays tissue repair. *Staphylococcus aureus*, *Escherichia coli*, and *Pseudomonas aeruginosa* are the main bacteria that colonize skin lesions [42], which can lead to local or systemic infection and/or microorganism resistance [46]. The mechanisms of action of BL in inhibiting bacterial growth have not been elucidated. A study that investigated BL delivered at 400–450 nm showed that it could inhibit the growth of *S. aureus*, *E. coli*, and *P. aeruginosa* strains, with the outcomes maintained for up to 48 h after the irradiation. The fluence of 6 J/cm^2^ proved to be effective in inhibiting these isolated bacteria [47].

**Figure 3:**
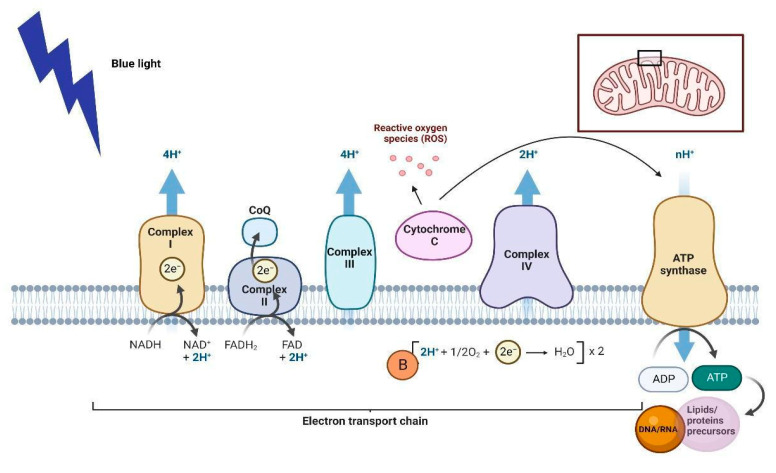
Mechanisms of action of blue light (BL). The effects of BL are dependent on light absorption by photoreceptors, stimulating mitochondrial function through cytochrome *c* oxidase in the mitochondrial membrane, leading to the production of adenosine triphosphate (ATP); reactive oxygen species (ROS); and precursors of lipids, proteins, and DNA/RNA. Created with BioRender.com (accessed on 11 January 2023).

According to published studies, photochemical reactions leading to the BL effects are dependent on light absorption by photoreceptors [48,49]. Cytochrome *c* oxidase (CCO) is known as the main photoreceptor in cells inducing NO and ROS production, and the activation of ion channels. Photoactivation can excite an electron within the CCO structure, stimulating the transfer of electrons through the respiratory chain. This leads to an increase in the electrochemical potential of the proton, increasing the activity of ATP synthase; cellular metabolism; and, consequently, the metabolic precursors of lipids, proteins, DNA, and RNA (Figure 3) [50]. Therefore, cell proliferation, migration, and adhesion are also increased, and cell apoptosis is prevented. Finally, NO can bind to CCO through the heme center. Photoactivation of CCO can dissociate NO, allowing increased respiration and greater ATP formation [50].

**Figure 4:**
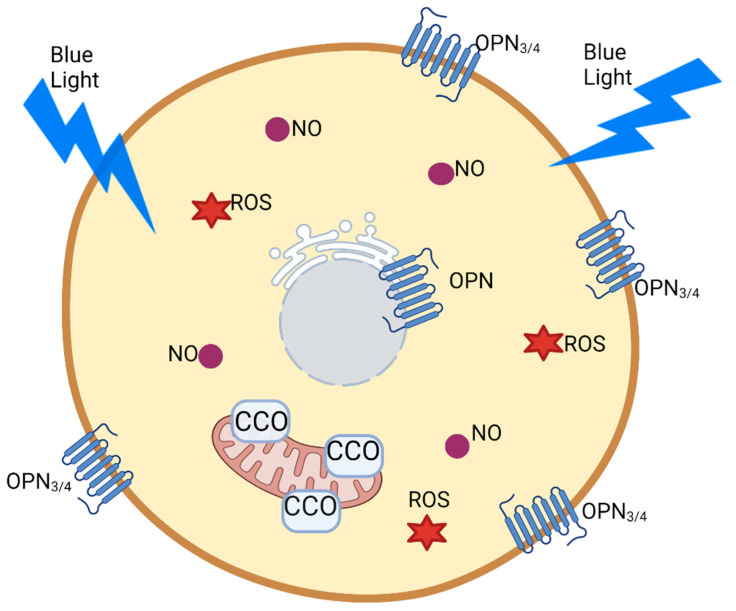
Photoreceptors present in skin cells (keratinocytes and melanocytes). Blue light can stimulate cytochrome *c* oxidase (CCO), increasing the production of adenosine triphosphate (ATP), nitric oxide (NO), and reactive oxygen species (ROS). Through opsin (OPN) signaling, blue light can regulate melanogenesis and vascular relaxation. Created with BioRender.com (accessed on 11 January 2023).

Recently, there has been increasing information regarding new molecular targets for photoactivation including a group of G-protein-coupled receptors called OPNs (Figure 4) [51]. There are four isoforms, namely OPN1 to OPN4, each with specific tissue expression. In humans, OPN1 and OPN2 (rhodopsin) are the main photoreceptors in the eye. In the skin, OPN4 regulates pigmentation through melanogenesis [52]. In addition, researchers investigated BL (400–440 nm) acting via OPN3 and OPN4 signaling and observed that BL induces relaxation in rat pulmonary arteries and in pulmonary arterial smooth muscle cells [53].

Despite the advance in the understanding of the mechanisms of action of BL, and the growing interest in the use of BL to reduce inflammation and limit bacterial growth in superficial tissues, additional studies are needed to expand the knowledge regarding the broad effects and optimal application parameters, with a focus on tissue repair. There has been no in depth evaluation of the literature concerning the action of the BL on wound healing. Hence, this review investigates the effect of BL on the healing process evaluating in vitro, preclinical, and clinical studies. It is expected that these findings will be of value to wound care specialists working in clinical research.

## 2. Methods

A scoping review was performed to explore and map the main findings available in the literature on the effects of BL in improving wound healing, summarizing their results and identifying potential gaps. This review was performed following the five stages described by Arksey and O’Malley (2005) [54], and updated by Levac et al. (2010) [55], which include: (1) identifying an initial research question, (2) identifying relevant studies, (3) study selection, (4) charting data, and (5) summarizing and reporting results. In addition, the Preferred Reporting Items for Systematic Reviews and Meta-Analyses Extension for Scoping Reviews (PRISMA-ScR) checklist was followed, to ensure it met reporting standards.

### 2.1. Stage 1: Identifying an Initial Research Question

This review aimed to understand the effects of BL on wound care in in vitro, preclinical, and clinical studies. The review question was thus elaborated using the PCC strategy: P (population), myofibroblasts, fibroblasts, keratinocytes, endothelial cells, and wounds in animal and human models (in vitro, preclinical, and clinical studies); C (concept), the use of BL in the healing process; and C (context), studies conducted in laboratories, hospitals, and at home. The review question adopted was: What are the effects of BL in in vitro, pre-clinical, and clinical studies on cutaneous wound healing?

### 2.2. Stage 2: Identifying Relevant Studies

Data collection was performed in the MEDLINE (PubMed), Web of Science, Scopus, and CINAHL databases in May 2022. The search strategies adopted are summarized in the Supplementary Material.

### 2.3. Stage 3: Study Selection

The articles included in this scoping review met the following eligibility criteria: (1) studies that evaluated the effect of BL (400–470 nm) on wound healing; and (2) studies in English, Spanish, or Portuguese published between 2012 and 2022. Reviews, systematic reviews, case reports, meta-analyses, letters to the editor, conference abstracts, editorials, protocols, and duplicate articles were excluded.

The search results were exported to Rayyan^®^ (Qatar Computing Research Institute, Doha, Qatar). A team member assigned the blind review role to two authors through the “Blind on” feature available in the app. The titles and abstracts of the pre-selected articles were read by two independent evaluators. After this step, the blinding feature was deactivated and possible conflicts in the evaluators’ decisions were resolved through analysis by a third evaluator.

### 2.4. Stage 4: Charting the Data

Data from the selected articles were extracted by two independent evaluators using a form created by the authors. These data included: (a) title, (b) author and year, (c) country of origin, (d) type of study, (e) type/time/wavelength (nm) of therapy, (f) distance from probe light, (g) energy density, (h) power, (i) power density, (j) time, (k) outcomes, and (l) conclusions. Data regarding the type of cells used were also collected for in vitro studies. In preclinical studies, information was added regarding the species used, and, in clinical and/or preclinical studies, the type of wound to which the therapy was applied. Discrepancies in the extracted data were discussed between the authors.

### 2.5. Stage 5: Summarizing and Reporting Results

The studies were grouped into tables according to the type of study (in vitro/mixed studies, preclinical studies, and clinical studies). Summarizing the findings in tables allowed comparison between the wound types and the key characteristics of the extracted data. The findings were then mapped and visualized using appropriate software, and a descriptive summary of the data is given, as recommended by Arksey and O’Malley (2005) [54]. A narrative summary of the findings as well as the effect they may have on future studies in this area is provided [55].

This study was registered at the Center for Open Science (OSF) registries (https://osf.io/brm3x. Accessed on 11 January 2023).

## 3. Results

**Figure 5:**
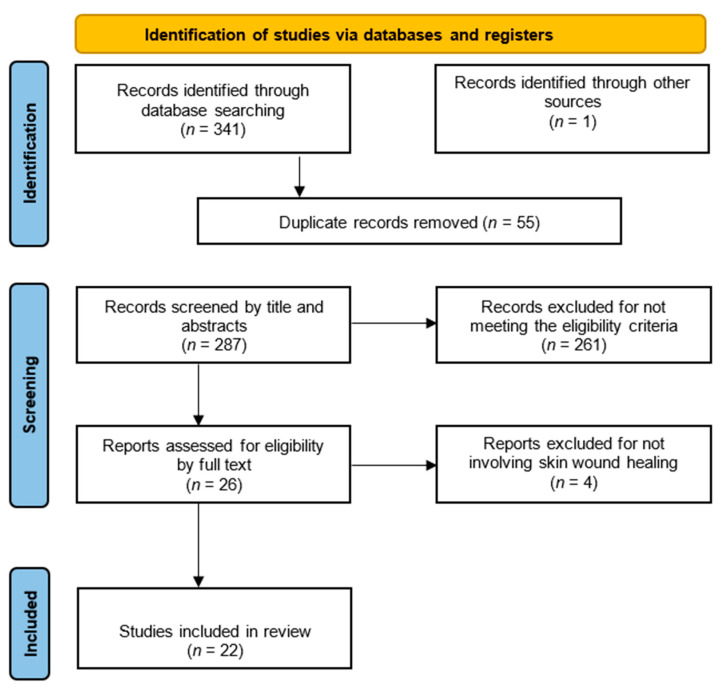
Preferred Reporting Items for Systematic Reviews and Meta-Analyses (PRISMA) flow diagram for inclusion or exclusion of studies used for this scoping review.

The initial search resulted in the identification of 342 articles, of which 341 were found by searching the databases and 1 article was identified by reading a previously selected study. After applying the eligibility criteria, 26 articles were selected, and having read all of them in full, four studies were excluded, resulting in 22 articles (Figure 5).

The contents were grouped into three categories to facilitate the presentation of the information extracted after reading and analyzing the recommendations: in vitro/mixed, pre-clinical, and clinical studies. Eight in vitro studies investigated the effects of BL exclusively in cell culture [56,57,58,59,60,61,62,63], two articles included in vitro and in vivo experiments [64,65], and one used ex vivo methods [66]. Immortalized myofibroblasts, fibroblasts, keratinocytes, endothelial cells [56,57,59,61,62,63,66], and primary cultures of human fibroblasts and keratinocytes [58,60] were used.

**Figure 6:**
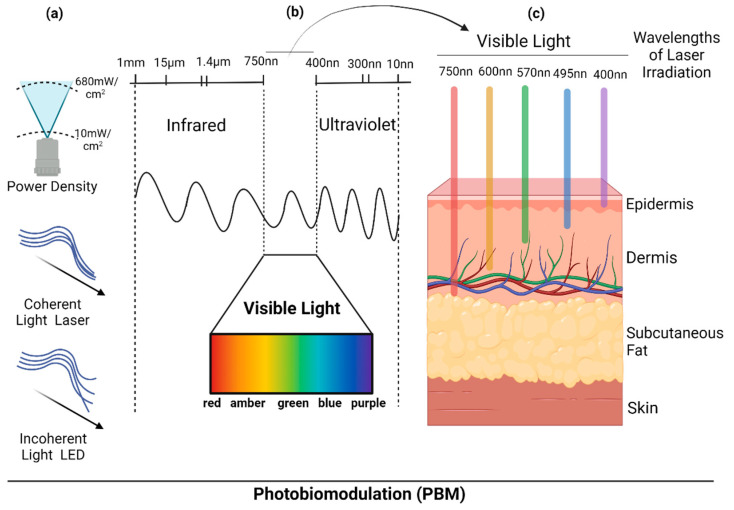
The parameters of the Photobiomodulation (PBM) used on the skin: (**a**) Graphical representation of the differences in the pattern of light emission; incoherent light emitted by the light emitting diode (LED); and coherent light emitted by the laser. (**b**) The wavelengths of visible and invisible light. (**c**) The light penetration into the epidermis and dermis, and the pattern of energy densities. Created with BioRender.com (accessed on 11 January 2023).

The study category, wavelength, power density, energy density, and level of evidence [67] of each study are summarized in Table 1. Figure 6 shows the PBM parameters used in the studies included in this review, such as the wavelengths and the dermal penetration of the visible light, the power density, and the difference between light-emitting diode (LED) and laser.

The power density (irradiance), specified in mW/cm^2^, ranged from 10 to 680 mW/cm^2^ in most studies. Ebrahiminaseri et al. (2021) [61] and Zhang et al. (2018) [64] did not describe the power density used in their studies. The authors also used different wavelengths (nm) and energy densities (J/cm^2^). The details for the studies are: 470 nm, 50 mW, and 30 J/cm^2^ [57]; 470 nm, 30 mW/cm^2^, and 3, 5, 10, and 55 J/cm^2^ [56]; 453 nm, 50 mW/cm^2^, and 2 and 30 J/cm^2^ [58]; 475 nm, 40 mw, and 24 J/cm^2^ [59]; 450 and 490 nm, 30 mW, and 30 J/cm^2^ [60]; 453 nm, 30 mW/cm^2^, and 2 and 30 J/cm^2^ [66]; 465 nm and 3 J/cm^2^, without a reported power density [64]; 450 nm, 75 mW/cm^2^, and 0.63 and 0.95 J/cm^2^ [61]; 420 nm, 680 mW/cm^2^, and 3.43, 6.87, 13.7, 20.6, 30.9, and 41.2 J/cm^2^ [63]; 460 nm, 4 mW/cm^2^, and 2.4 and 4.8 J/cm^2^ [65]; and 400–450 nm, 120 mW/cm^2^, and 28.9 J/cm^2^ [62].

Another variable considered important for adequate light delivery to the tissues is the irradiation time. Among the seven in vitro/mixed articles, the irradiation time ranged from 5 s to 20 min (Table 2) [57,59,61,62,63,64,65]. Three studies did not report the irradiation time [58,60,66], and one study reported that the time was given by the equipment automatically [56].

Studies that investigated BL with an energy density of <20 J/cm^2^ described better results in the healing process. A study using 2 J/cm^2^ observed stimulation of fibroblast activity, as well as an improvement in cell metabolic processes and increased collagen production [58]. Another study that used an energy density of 5 J/cm^2^ suggested that BL has an anti-inflammatory action by decreasing the expression of interleukin 6 (IL-6) (*p* < 0.0001) [56], and another described the decrease in tumor necrosis factor α (TNF-α) (*p* < 0.0001) and IL-6 (*p* < 0.0001) when treated with BL at 450 nm and 0.63 J/cm^2^ [61]. The authors also described increased proliferation and migration of fibroblasts and increased expression of GFs like TGF-β (*p* < 0.0001) and VEGF (*p* < 0.01). The ex vivo results, using the parameters of 453 nm and 2 J/cm^2^, showed an increase in OPN3 expression leading to improvement in lesion closure [66]. Rossi et al. (2021) [63] reported that fibroblasts had increased metabolic activity, and both fibroblasts and keratinocytes showed increased migration during the scratch assay.

Cai et al. (2022) [65] demonstrated a higher concentration of NO in the combined BL/RL group compared with the control group (*p* < 0.01). The NO concentration in the BL group was significantly higher than in the RL group (*p* < 0.05). Cell migration in the BL group was also significantly lower than in the RL (*p* < 0.05) and BL/RL groups (*p* < 0.05). In diabetic rats, the use of combined light significantly accelerated the healing process (*p* < 0.01), with increased angiogenesis and collagen deposition [63]. Another study also evaluated the combined effect of BL/RL (425 nm and 3 J/cm^2^; 465 nm and 3 J/cm^2^, respectively) and compared it with RL [64]. The authors demonstrated that there was no effect on cell growth and migration in human fibroblasts compared with the RL group; however, BL was more effective in healing the excisional wound in vivo compared with RL. On the other hand, in vitro studies that used an energy density between 20.6 to 55 J/cm^2^ showed greater cellular apoptosis/necrosis and inhibitory and cytotoxic alterations for myoblasts, fibroblasts, keratinocytes, and endothelial cells [56,57,60,63,66], and reduced proliferation and migration of keratinocytes [62]. Several genes involved in the TGF-β signaling pathway were also inhibited, as well as transcripts involved in collagen production [60].

Seven studies were included in the preclinical category (Table 3) [68,69,70,71,72,73,74], and the wavelength (nm) of BL ranged from 405 to 470 nm. One study used equipment with combined BL/RL (470 and 685 nm, respectively) [71]. The energy density used in these studies ranged from 1.5 to 30 J/cm^2^. Two studies did not specify the energy density used [68,70]. One study did not describe power density [74], and the others fluctuated from 0.008 to 200 mW/cm^2^. The treatment time varied from 3 to 28 days, and the duration of BL application was from 25 s to 1 h. Two studies used Wistar rats [73,74], and three used Sprague Dawley rats [71] and rabbits [72]. Two studies investigated a third-degree burn model [73,74], two studies investigated excisional injuries [68,72], one study investigated an ischemia model [69], one study investigated a skin abrasion model [70], and one study investigated a sutured incision model [71]. Only two studies utilized a power of 1000 mW [70,74].

De Alencar Fernandes Neto et al. (2019) [74] investigated the action of BL in a third-degree burn model using BL at 470 nm and 12.5 J/cm^2^ and observed a proliferation of blood vessels on day 7 post-injury (*p* = 0.01); however, there was no significant regression of the wound area. Fekrazad et al. (2017) [73] used BL at 405 nm and 1.5 J/cm^2^, comparing three different wavelengths (532, 660, and 810 nm). They observed a reduction in the lesion area compared with the control (*p* < 0.05), without significant improvement in the inflammatory phase, and complete formation of all skin appendages, even after 21 days of treatment.

In the studies with an excisional wound model, Cheon et al. (2013) [68] used combined BL/RL and green light and observed that BL promoted a better healing rate (*p* < 0.05) and an increase in collagen fibers compared with the control, but this was lesser than BL. Y. Li et al. (2016) [72] used a combined BL/RL and investigated excisional wound healing in the ears of white rabbits treated with BL. They observed that on days 16 and 17, after daily treatment for 15 or 30 min, the healing rates in BL groups were 25% and 37.5%, respectively, while in the control group, it was 12.5%. Collagen fiber was diminished, and the epidermis was reduced in thickness. Based on immunostaining, there was lower expression of EGF (*p* < 0.05), FGF (*p* < 0.05), cluster of differentiation 31 (CD31) (*p* < 0.001), and Ki67 (*p* < 0.001) in the BL group after 21 days of light application. The effect of BL on wound healing in this study was lower than in the RL group (630 nm). 

Figurová et al. (2016) [71] investigated combined light treatment (470/629 nm) in a sutured incision model. They demonstrated that the treatment accelerated reepithelization and the formation of cross-linked collagen fibers compared with sham irradiated control wounds.

In an ischemia model, Dungel et al. (2014) [69] observed that on day 3, the necrotic area in the BL group (470 nm) had decreased by 11% compared with the control group. The perfusion was better, and the number of α-smooth muscle actin (α-SMA)-positive cells, a marker of myofibroblasts, was higher in RL (629 nm) when compared with BL. On day 7, BL showed significant effects on wound healing, such as a 13% higher perfusion rate compared to BL, and the number of α-SMA-positive cells was similar between the groups. 

Cicchi et al. (2016) [70] studied abraded lesions. They applied two wavelengths of BL (410 and 435 nm) and observed that both light lengths led to a 50% reduction in the cellular inflammatory infiltrate. The epidermis was completely regenerated in both groups, as evidenced by the thickness of the epidermis and the similar density of keratinocytes in the injured area compared with the control. 

Five studies were included in the clinical category (Table 4) [75,76,77,78,79], totaling 64 patients with wounds of different etiologies that were evaluated before and after light application. One of these studies evaluated the effect of BL on 12 patients; the control group comprised 8 patients who were not irradiated [79]. There was great variety in the wound etiologies, such as patients with sclerotic skin ulcers on the fingers and hands [79], venous ulcers [75], peripheral arterial disease [76], venous stasis ulcer [76], post-traumatic skin lesions [77], cutaneous vasculitis [76], wound dehiscence [76], and second-degree thermal burns [78]. The mean age of the participants was 68.42 years, although one study did not report the age of the participants [78]. Most studies used the EmoLED^®^ device (400–430 nm at 7.2 J/cm^2^, with a power density of 120 mW/cm^2^), and one study [78] used a biophotonic platform consisting of a chromophore-containing gel and a multi-LED lamp delivering BL with wavelengths ranging from 440 to 460 nm, and a power density between 55 and 129 mW/cm^2^. In three studies [76,77,78], light was applied once a week for 60 s.

The follow-up period varied among the studies. Dini et al. (2022) [77] treated venous leg ulcers and traumatic ulcers once a week for 4 weeks. Vasculitis was treated with two consecutive applications of the BL twice a week for 4 weeks. Spinella et al. (2022) [79] treated the ulcers for 8 weeks, and Marchelli et al. (2019) [76] treated them for 10 weeks. Mosti and Gasperini (2018) [75] followed three patients in their study and presented different follow-up periods. For one patient, there were 11 applications in 21 weeks, the second patient had 5 applications in 12 weeks, and the third had 8 applications in 9 weeks. Mallergaard et al. (2021) [78] evaluated patients with acute second-degree burns and performed a pilot study using an ex vivo human skin model. Patients were treated twice per week for 2–3 weeks, and the biopsy punches were divided into three groups: untreated control, light-emitting diode (LED; BL alone), and gel and BL, with a lamp at a distance of 5 cm for 9 min.

Spinella et al. (2022) [79] observed complete healing of the lesions in 41.6% of cases during follow-up and treatment with BL. After the 8th week of treatment, there was a decrease in the lesion area, reepithelialization of the wound bed with the formation of granulation tissue, and an improvement in pain, compared with the beginning of treatment. Lesions in the control group treated with systemic therapy and conventional local therapy showed only a reduction in the size of the lesion and a trend for an area reduction at the end of the follow-up.

In their case series, Marchelli et al. (2019) [76] demonstrated that 16 of 19 patients (84%) with wounds that received BL treatment responded to treatment, reaching an average of 50% reepithelialization within the maximum observation period of 10 weeks. Five wounds achieved between 50% and 80% reepithelialization, and six wounds achieved >80% reepithelialization. Among these, half reached total reepithelialization in an average of 7 weeks. In the group of patients with venous ulcers, seven of nine responded to treatment, with an average reepithelialization of 65%. In two cases, there was no response to treatment; both patients had a diagnosis of diabetes. In the three post-traumatic ulcers evaluated, all responded to treatment, reaching an average of 77% reepithelialization. The wounds of three patients diagnosed with vasculitis responded to treatment with a mean of 43% reepithelialization. One of two patients with peripheral arterial disease lesions responded to treatment. The patient with wound dehiscence responded to treatment (85% reepithelialization). In summary, almost all treated lesions responded to BL treatment during the 10-week follow-up period, and there were no adverse events.

Dini et al. (2022) [77] reported an average healing rate of 0.098 mm/day for venous leg ulcers, 0.353 mm/day for traumatic ulcers, and 0.09 mm/day for vasculitis. Overall, 16 patients had a reduction in wound size, two patients were completely healed, and two patients did not improve in terms of area reduction. Mellergaard et al. (2021) [78] demonstrated that in all cases complete healing was observed without the need for skin grafting, and there were no adverse events related to the biophotonic platform. The described healing time extended from 7 days for some partial-thickness burns and continued to a maximum of 21 days for full-thickness burns. In a pilot study using an ex vivo human skin model, the treatment led to an increase in collagen I, FGF2, anti-inflammatory cytokines, TGF-β1, and TGF-β3.

Mosti and Gasperini (2018) [75] followed three patients with chronic ulcers. The first was a patient with a chronic venous ulcer who received five sessions of BL during a 12-week period. There was an improvement in the appearance of the wound bed, a reduction in its depth, and revitalization of the perilesional skin. The second was a patient with a history of lower limb revascularization and multiple skin grafts on both legs. After two BL applications, the lesions of the right limb closed completely. In the left limb, lesions with bone exposure were submitted to 11 applications of BL in a period of 21 weeks leading to a wound reduction of 90%. The third patient had venous ulcers in both lower limbs for 7 years, with a history of skin grafts from dermal and autologous substitutes treatment that did not solve the problem. The therapeutic protocol included weekly dressings, compression therapy, and BL treatment that was performed eight times in 9 weeks. Healing was achieved for the right limb ulcer after 4 weeks. Half of an ulcer in the left limb was treated with BL, the other half with just the standard dressing. After 9 weeks, the part that received the BL application showed 95% reepithelialization, and the other part of the limb achieved 80% reepithelialization.

## 4. Discussion

Based on this review, most published research on the effectiveness of BL in wound healing has been carried out in animal models and in cell cultures [56,57,58,59,60,61,62,63,64,65,66,68,69,70,71,72,73,74]. There are advantages to conducting experiments in these models because many variables can be controlled more easily; this factor may explain the greater number of this type of study. However, there are morphological and molecular differences in experiments conducted using in vitro and in vivo models that prevent translation to humans [80,81]. There was also a great variety of device parameters used among the studies, such as the wavelength, power (specified in mW for laser therapy), power density (irradiance), time of irradiation (combined with power or irradiance), energy, and energy density, which may have contributed to the variety of the findings. In certain circumstances, the authors did not report all the parameters they used [58,60,61,64,66,68,70]. Conversely, in other studies, the parameters were given automatically by the equipment [56], instead of being chosen specifically to replicate data from a previous study. This lack of quality control among so many studies created a limitation that did not allow us to conclude on the best parameters to improve tissue repair.

Despite the great variability in parameters observed in this review, we suggest that BL provided at a low energy density (<20 J/cm^2^) is able to decrease pro-inflammatory cytokines and stimulate GFs, improving cell migration/proliferation and angiogenesis. In addition, BL provided at <20 J/cm^2^ could increase collagen production and decrease the wound area [65,69,70,71,73,74]. It is worth noting, however, that some of these data are contradictory. Fekrazad et al. (2017) [73] observed no significant modulation in inflammatory cytokine, while Y. Li et al. (2016) [72] observed decreases in collagen deposition, the thickness of the epidermis, important GFs, and cell proliferation markers.

On the other hand, studies have demonstrated that BL provided at a high energy density (20.6–50 J/cm^2^) leads to cellular apoptosis during tissue repair, inhibitory and cytotoxic changes in fibroblast cultures, lower GF concentrations (such as TGF-β1), and the inhibition of procollagen [60]. These data support previous studies demonstrating that depending on the lighting parameters, BL can promote a reduction in the production of inflammatory cells, cell proliferation and migration, and the synthesis of collagen, angiogenesis, and granulation tissue [82,83,84].

Our evaluation of the cellular and molecular mechanisms that could lead to the changes observed after BL irradiation suggests that there is a gap in this information. Only Cai et al. (2022) [65] addressed this issue. They studied photoreceptors and their cellular and molecular actions. They observed increased OPN expression and suggested that it might improve NO production, reduce ROS, and have a positive effect on wound healing in the scratch assay. It has been well established that to induce biological activity, light must be absorbed by photoreceptors, promoting their change into an excited state; these molecules then stimulate secondary targets in the cell, transducing the light signal into a physiological response [85,86]. NO regulates several wound-healing processes, including inflammation, cell proliferation, collagen formation, and angiogenesis, and it has an antimicrobial action through ROS, which is involved in fighting invading microorganisms and signaling cell survival [87]. The results of such studies are important, although stronger evidence based on clinical studies is required.

In general, there are relatively few clinical studies in the literature [75,77,78,79]. This may be due to the difficulties associated with conducting clinical studies. Well-controlled and blinded studies face problems such as the presence of many internal and external variables, including wound size, patient comorbidities, and concomitant treatments [88,89]. The studies were carried out in patients who had ulcers of various etiologies, from acute [75,78] to chronic wounds, in which advanced treatments for wound healing, such as skin grafts, had been performed [75]. Moreover, most studies did not have a control group, using only the results of follow-up treatment. The lack of an adequate control group and the great heterogeneity of the variables may prove to be an important bias, requiring further studies with more robust evidence [88,89]. Unlike in vitro and preclinical studies, however, clinical studies mostly used the same device parameters [75,77,79]. However, most of them were conducted using non-coherent light sources such as light-emitting diodes (LEDs). We believe that the researchers chose these LED-based devices because they are cost-effective, easy to configure/manipulate, and allow for the emission of two or more wavelengths of light in a single application. Nevertheless, there is controversy over which type of therapy is most effective. Lasers have well-defined properties such as monochromaticity (wavelength with a narrow spectral band), coherence (phase waves) and collimation (parallel waves), while the LEDs despite having similar physical properties, have less specificity [90].

The use of similar parameters, in a way, gave credibility to the data found, and despite the presence of many internal and external variables, we were able to conclude that BL irradiation improves the healing process presented in clinical studies by closing the lesion in a follow-up time shorter than the time the wound remained open.

The main limitation of this review is the potential publication bias in that studies may have been missed even with the extensive literature searches. In addition, studies in languages other than English and studies published more than 10 years ago were not evaluated. In addition, the lack of standardization of the parameters used in the light-emitting devices made it difficult to interpret the results in several of the analyzed studies. Finally, clinical studies used small samples with wounds of different etiologies and low levels of evidence, and there was a lack of a standardized protocol.

## 5. Challenges and Future Perspectives

People with skin lesions face pain, discomfort, changes in body image, reduced mobility, and an inability to perform daily tasks, all of which directly impact their quality of life. Several strategies have been investigated to lead to rapid and effective tissue repair, to decrease treatment times, and to improve the prospects of quality of life. BL therapy is a treatment that contributes to the healing process and modulates cell signaling, especially by activating mitochondrial proteins, inducing ATP production, increasing ROS, and activating the OPN signaling pathways. Determining the best parameters of the equipments to produce an effective response in tissue repair is a challenge for researchers and health professionals, and the lack of standardized protocols limits the understanding of the best therapeutic response. In addition, the small number of preclinical studies makes it difficult to fully understand the molecular and cellular mechanisms of PBM. Understanding how light is absorbed by cells and tissues and all its effects is necessary to direct future clinical and multicenter studies, and to standardize protocols to provide safe and effective adjuvant treatment to people.

## 6. Conclusions

BL at a low energy density (<20 J/cm^2^) stimulates different cell types and proteins involved in healing. Conversely, BL at a high energy density, 20.6–50 J/cm^2^, significantly reduces cell proliferation, migration, and metabolism. Thus, BL irradiation must be carefully evaluated before establishing its use in clinical practice. Additional studies are needed to contribute to the knowledge of the appropriate parameters of light to be used, and the cellular and molecular mechanisms by which BL improves the wound-healing process [50]. It is worth mentioning that BL is an adjuvant treatment and it is necessary to follow the established guidelines regarding hygiene/debridement, moisture balance, and the application of adequate coverage.

## Figures and Tables

**Table 1:** Summary of the main parameters used in the studies compiled in the review.

Study Category	Reference	Wavelength (nm)	Power Density (mW/cm^2^)	Energy Density (J/cm^2^)	Level of Evidence
In vitro/mixed studies	Teuschl et al., 2015 [57]	470 and 630	50	30	5
	Masson-Meyers et al., 2016 [56]	470	30	3, 5, 10, and 55	5
	Mignon et al., 2016 [58]	453	50	2 and 30	5
	Rohringer et al., 2017 [59]	475	40	24	
	Castellano-Pellicena et al., 2018 [66]	453	30	2 and 30	5
	Mignon et al., 2018 [60]	490	30	30	5
	Zhang et al., 2018 [64]	465	Not described	3	
	Ebrahiminaseri et al., 2021 [61]	450	75	0.63 and 0.95	5
	Rossi et al., 2021 [63]	420	680	3.43, 6.87, 13.7, 20.6, 30.9, and 41.2	
	Cai et al., 2022 [65]	460	4	2.4 and 4.8	5
Pre-clinical studies	Cheon et al., 2013 [68]	470	3.55	Not described	5
	Dungel et al., 2014 [69]	470	50	30	5
	Cicchi et al., 2016 [70]	410 and 435	1.27	Not described	5
	Figurová et al., 2016 [71]	475	0.0008	3.36	5
	Li et al., 2016 [72]	460	50	45.9	5
	Fekrazad et al., 2017 [73]	405	200	1.5	5
	Neto et al., 2019 [74]	470	Not described	50	5
Clinical studies	Mosti & Gasperini, 2018 [75]	415	120	7.2	4
	Marchelli et al., 2019 [76]	420	120	7.2	4
	Dini et al., 2021 [77]	405	120	Not described	4
	Mellergaard et al., 2021 [78]	440	55 and 129	Not described	4
	Spinella et al., 2022 [79]	400	120	7.2	4

Table note: cm^2^: square centimeter; nm:nanometer; mW/cm^2^: milliwatts per centimeter squared; J: joules/cm^2^.

**Table 2:** Summary of the in vitro/mixed studies compiled in the review.

Title	Author and Year	Country	Study Type	Therapy Type/Wavelength (nm)	Distance from Probe Light	Energy Density (J/cm^2^)	Power	Power Density (mW/cm^2^)	Time
Phototherapy with LED light modulates healing processes in an in vitro scratch-wound model using 3 different cell types	Teuschl et al., 2015 [57]	Austria	In vitro: Myoblasts, fibroblasts, and keratinocytes	LED/470 or 630	10 cm	30	N.D.	50	10 min/day for 5 consecutive days
Outcomes measured Scratch wound assay Proliferation assay Apoptosis/necrosis rate			Conclusion BL decreased myoblast and keratinocyte proliferation. BL prevented any bridging of the gaps for 6 weeks in all cell types. BL increased the percentage of apoptotic cells in all cell types. BL induced necrosis in myoblasts and fibroblasts.						
Blue light does not impair wound healing in vitro	Masson-Meyers et al., 2016 [56]	USA	In vitro: Human dermal fibroblasts	LED/470	1–2 mm	3, 5, 10, and 55	150 mW	30	Automatic timed
Outcomes measured Scratch wound assay Cell migration Wound closure analysis Protein synthesis			Conclusion Cells irradiated at 55 J/cm^2^ had slower wound closure, slower migration, and lower viability. Cells irradiated at 5 J/cm^2^ had an increase in total protein synthesis. After irradiation, there was a dose-dependent decrease in the IL-6 concentration.						
Photobiomodulation of distinct lineages of human dermal fibroblasts: a rational approach towards the selection of effective light parameters for skin rejuvenation and wound healing	Mignon et al., 2016 [58]	United Kingdom	In vitro: Primary human reticular and papillary dermal fibroblast	LED/453–850	N.D.	0.1–100	N.D.	10 and 50	N.D.
Outcomes measured Scratch wound assay Proliferation assay Metabolic activity assay Collagen assay			Conclusion A low dose of BL stimulated the metabolic activity of dermal fibroblasts. A low dose of BL induced cell metabolic activity. A high dose of BL stimulated the metabolic activity of reticular fibroblasts and inhibited papillary fibroblasts. Low doses of BL increase collagen production.						
The impact of wavelengths of LED light-therapy on endothelial cells	Rohringer et al., 2017 [59]	Austria	In vitro: Endothelial cells	LED/475, 516, and 635	2 cm	24	N.D.	40	10
Outcomes measured Proliferation and MTT assays 2D migration: scratch assay 3D migration: bead assay Quantification of fluorescent cells and vascular networks Protein expression ROS formation			Conclusion BL decreased metabolic activity and showed no effect on the proliferation of endothelial cells. BL induced ROS formation. BL did not affect cell migration. BL upregulated dipeptidyl peptidase IV, neuregulin 1-B1, and placenta growth factor.						
Differential response of human dermal fibroblast subpopulations to visible and near-infrared light: the potential of photobiomodulation for addressing cutaneous conditions	Mignon et al., 2018 [60]	United Kingdom	In vitro: Primary human reticular and papillary dermal fibroblast	LED/450, 490, 550, 590, 650, and 850	N.D.	0–250	N.D.	30	Once a day for 3 consecutive days (no information on the daily irradiation time)
Outcomes measured ROS release/detection Mitochondria tracker Metabolic activity assay Collagen assay			Conclusion BL had a cellular inhibitory effect at low- to mid-dose levels (<30 J/cm^2^), and it was cytotoxic at higher levels (>30 J/cm^2^). BL was associated with the induction of intracellular ROS in dermal fibroblasts. Reticular and papillary DF exhibited different responses in gene expression pathways related to cellular metabolism and protein synthesis when irradiated with BL. BL at a low or high dose decreased mRNA expression of genes involved in collagen production.						
Does blue light restore human epidermal barrier function via the activation of opsin during cutaneous wound healing?	Castellano-Pellicena et al., 2018 [66]	Netherlands	Ex vivo: Human skin model In vitro: Fibroblasts and keratinocytes	LED/ex vivo: 453 or 656; keratinocytes: 447, 505, 530, 655, and 850	N.D.	453 nm: 2 J/cm^2^ daily 656 nm: 30 J/cm^2^ daily	N.D.	30–50	N.D.
Outcomes measured Expression of opsins in human skin Metabolic assay Migration assay Wound closure rate Scratch wound assay			Conclusion BL induced OPN3 expression. A low dose of BL (2 J/cm^2^) increased the rate of wound closure. BL increased keratinocyte metabolic activity at 2 J/cm^2^. However, at 30 J/cm^2^ there was no effect. There were no changes in cell morphology. DNA synthesis was reduced, while differentiation was induced. BL at 2 J/cm^2^ did not affect keratinocyte migration in a scratch-wound assay, but BL at 30 J/cm^2^ reduced keratinocyte migration.						
LED phototherapy with gelatin sponge promotes wound healing in mice	Zhang et al., 2018 [64]	China	In vitro: Fibroblasts In vivo animal model: BALB/c mouse excisional wound	LED/465 and 625	15 cm	3	21 W	N.D.	10 min
Outcomes measured Cell growth and migration Wound healing rate			Conclusion Wounds in the BL group reduced fastest. BL promoted cell growth.						
Combination treatment of dendrosomal nanocurcumin and low-level laser therapy develops proliferation and migration of mouse embryonic fibroblasts and alters TGF-β, VEGF, TNF-α and IL-6 expressions involved in the wound healing process	Ebrahiminaseri et al., 2021 [61]	Iran	In vitro: Mouse embryonic fibroblasts	LED/450	6 cm	0.63 and 0.95	75 mW	N.D.	224 and 337 s
Outcomes measured MTT assay Real-time PCR ELISA Real-time PCR Cell cycle analysis by flow cytometry			Conclusion Treatment with dendrosomal nanocurcumi and 0.95 J/cm^2^ dose of LLLT induced cell proliferation and migration and regulated intracellular ROS accumulation. BL at doses of 0.63 and 0.95 J/cm^2^ increased TGF-β expression, and doses of 0.10 and 0.95 J/cm^2^ increased VEGF expression. BL decreased the expression of TNF-α and IL-6.						

Table note: cm^2^: square centimeter; J: joules; LED: light-emitting diode; LLLT: low level laser therapy; nm: nanometer; mW/cm^2^: milliwatts per centimeter squared; N.D.: not declared.

**Table 3:** Summary of the preclinical studies compiled in the review.

Article Title	Author and Year	Country	Animal	Wound Etiology	Therapy Type/Wavelength (nm)	Distance from Probe Light	Energy Density (J/cm^2^)	Power	Power Density (mW/cm^2^)	Time
Low-level light therapy by red green–blue LEDs improves healing in an excision model of Sprague–Dawley rats	Cheon et al., 2013 [68]	Republic of Korea	Sprague-Dawley rats	Excisional	LED/470, 525, and 633	N.D.	N.D.	N.D.	3.55 (blue), 4.02 (green), 6.78 (red)	1 h/ day for 9 days
Outcomes measured Immunohistochemical analysis			Conclusions All irradiated groups showed a decreasing rate of defect size quicker than the none-irradiated group. Masson’s trichrome staining showed more collagen in the order of the green, the red, the blue, and no irradiated group. No significant difference for PCNA expression between the irradiated and non-irradiated groups.							
Low-level light therapy by LED of different wavelengths induces angiogenesis and improves ischemic wound healing	Dungel et al., 2014 [69]	Austria	Sprague-Dawley rats	Ischemic	LED/470 and 629	10cm	30		50	10 min for 5 days
Outcomes measured Area of necrosis Laser Doppler imaging Immunohistochemical analysis			Conclusions On day 3, the necrotic area in the RL group was reduced by 29% and BL was decreased by 11% compared with the control group. The RL group had better perfusion and more α-SMA-positive cells. On day 78, RL and BL showed significant effects on wound healing. At this point, the BL group showed a 13% higher perfusion rate than the RL group, and the number of α-SMA-positive cells was similar.							
Histological assessment of a combined low-level laser/light-emitting diode therapy (685 nm/470 nm) for sutured skin incisions in a porcine model: A short report	Figurová et al., 2016 [71]	Slovak Republic	Minipigs	Sutured incisions	LED/470 and 629	N.D.	3.36	N.D.	0.008	420 s
Outcomes measured Histological evaluation			Conclusions On day 3, the incisions in the control group were not completely bridged by epithelial cells and the tissue had fewer fibroblasts. The inflammatory phase was almost complete, and the layer of the dermis without a significant quantity of dermis layer was without a significant quantity of collagen in both groups. The incisional gap had layers of keratinocytes and most fibroblasts were oriented horizontally. Crosslinked collagen fibers predominated, indicating progressive scar formation.							
The histopathological investigation of red and blue light emitting diode on treating skin wounds in Japanese big-ear white rabbit	Li et al., 2016 [72]	China	Big-ear white rabbits	Excisional	LED/460 and 630	15 cm	45 and 90	N.D.	50	15 or 30 min for 21 days
Outcomes measured Calculation of the number and surface area of healing wounds Immunohistochemical and Masson staining			Conclusions The BL groups showed a reduction in wound size beginning 2–3 days after starting treatment and had a prolonged healing time compared with the RL groups. On day 2, the group that received 15 min of BL had a more healed area than the group that received 30 min of BL. The effect of BL was poorer than the RL because RL promoted proliferation of fibroblasts, vascular endothelial cells, and epidermal cells.							
Observation of an improved healing process in superficial skin wounds after irradiation with a blue-LED hemostatic device	Cicchi et al., 2016 [70]	Italy	Sprague-Dawley rats	Abrasions	LED/410 and 435	1 cm	N.D.	1 W	1.27	25 s
Outcomes measured Immunohistochemical analysis Morphometry			Conclusions The BL group showed more collagen, a better-recovered morphology, and organized collagen with a minimal inflammatory response on day 8 after the wound.							
Evaluation of therapeutic laser influences on the healing of third-degree burns in rats according to different wavelengths	Fekrazad et al., 2017 [73]	Iran	Wistar rats	Third-degree skin burns	Laser/405, 532, 660, and 810	1 mm	1.5	N.D.	200	N.D.
Outcomes measured Wound contraction Histological evaluation			Conclusions All laser groups showed better wound contraction compared with the control group, but these differences were not significant except between the RL and BL groups on day 21. Histological evaluation revealed that inflammation and fibrous and granulation tissues were observed in all groups on 21 days.							
Effect of blue LED on the healing Process of third-degree skin burns: clinical and histological evaluation	Neto et al., 2019 [74]	Brazil	Wistar rats	Third-degree skin burns	LED/470	In contact with the wound	12.5 or 50	1 W	N.D.	28 s
Outcomes measured Wound retraction index (WRI) Histological evaluation			Conclusions There were no significant differences in the WRI between the BLUE and control groups on days 7, 14, 21, and 28 days after wounding. On day 7, all animals treated with BL had wound reepithelialization. On day 7, there was a significant increase in the angiogenic index in the BLUE group compared with the control group. At the other evaluation times, there were no differences between the groups.							

Table note: cm^2^: square centimeter; J: joules; LED: light-emitting diode; LLLT: low level laser therapy; nm: nanometer; mW/cm^2^: milliwatts per centimeter squared; ND: not declared; PCNA: proliferating cell nuclear antigen.

**Table 4:** Summary of the clinical studies compiled in the review.

Title	Author and Year	Country	Wound Etiology/Localization	Therapy Type/Wavelength (nm)	Distance from Probe Light	Energy Density (J/cm^2^)	Power	Power Density (mW/cm^2^)	Time
Observations made on three patients suffering from ulcers of the lower limbs treated with blue light	Mosti & Gasperini, 2018 [75]	Italy	Different etiologies: one venous ulcer, one peripheral arterial disease, and one venous stasis ulcer	EmoLED^®^/400-430	4 cm	7.2	N.D.	120	60 s
Outcomes measured Clinical observations were made regarding wound size, bed, depth, and perilesional skin				Conclusions BL increased the wound healing rate					
Photobiomodulation with blue light in non-healing wounds: Case series evaluation	Marchelli et al., 2019 [76]	Italy	Different etiologies: 11 venous ulcers, six post-traumatic skin lesions, three cutaneous vasculitis, one wound dehiscence, and four peripheral arterial disease	EmoLED^®^/400–430	N.D.	7.2	N.D.	120	The 60 s/once a week
Outcomes measured Clinical observations were made regarding wound size, bed, depth, and perilesional skin				Conclusions Of the 19 wounds observed, 84% responded to the treatment, reaching an average of 50% reepithelization within the maximum 10-week observation period. Five wounds reached 50%–80% reepithelization and six wounds reached over 80% reepithelization. In conclusion, BL contributed significantly to the healing process of the wounds observed.					
Blue light emission in the management of hard-to-heal wounds	Dini et al., 2021 [77]	Italy	Different etiologies: 12 venous ulcers, six cutaneous small vessels vasculitis, and two traumatic ulcers	EmoLED^®^/400-430	4 cm	7.2 J/cm^2^	N.D.	120	60 s once a week
Outcomes measured Clinical observations were made regarding wound size, bed, perilesional skin, pain scoring and healing rate				Conclusions Sixteen patients had a reduction in wound size, two patients were completely healed, and two showed no improvement. Almost all patients showed pain reduction, and all the patients increased wound bed scores.					
Energy for the treatment of acute second-degree burns	Mellergaard et al., 2021 [78]	France and Italy	18 patients with second-degree thermal burns/ex vivo human skin model	LED/440 and 460	5–9 cm	N.D.	N.D.	55 and 129	Group 1: twice a week for 2–3 weeks for 5 min Group 2: 2–3 times a week for 5 min
Outcomes measured Quantification of collagen I human genome array				Conclusions Complete wound healing in all participants. There was an increase in collagen I after the treatment with gel plus blue LED on ex vivo human skin model. The expression of FGF2 and the anti-inflammatory cytokines TGF-β1 and TGFβ3 was induced by the treatment					
Photobiomodulation therapy: A new light on the treatment of systemic sclerosis skin ulcers	Spinella et al., 2022 [79]	Italy	12 patients with sclerotic ulcers on finger and hands	EmoLED^®^/4 00-430	4cm	7.2 J/cm^2^	N.D.	120	The 60 s/once a week
Outcomes measured Wound area, length, and width Wound bed characteristics Pain Exudate characteristics				Conclusions BL produced faster healing with the formation of granulation tissue and regularization of wound margins compared to the controls treated with standard therapies					

Table note: cm^2^: square centimeter; J: joules; LED: light-emitting diode; LLLT: low level laser therapy; nm: nanometer; mW/cm^2^: milliwatts per centimeter squared; ND: not declared.

## Data Availability

The data available at REDU (https://redu.unicamp.br, accessed on 15 January 2023).

## Supplementary Materials

The following supporting information can be downloaded at: https://www.mdpi.com/article/10.3390/life13020575/s1, Database formulas used during literature search.
