# Supplementary material for: Photobiomodulation with Blue Light on Wound Healing: A Scoping Review
Source: Life (Basel). 2023 Feb 18;13(2):575. doi: 10.3390/life13020575 (PMC9959862; doi:10.3390/life13020575)
Supplement: Supplementary file 1 [file life-13-00575-s001.zip › life-2228613-supplementary.pdf]

Medline Search Formula

1. ((Low-Level Light Therapy[MeSH Terms]) OR (Light Therapies, Low-Level)) OR (Light Therapy, Low-Level)) OR (Low Level Light Therapy)) OR (Low-Level Light Therapies)) OR (Therapies, Low-Level Light)) OR (Therapy, Low-Level Light)) OR (Photobiomodulation Therapy)) OR (Photobiomodulation Therapies)) OR (Therapies, Photobiomodulation)) OR (Therapy, Photobiomodulation)) OR (LLLT)) OR (Laser Therapy, Low-Level)) OR (Laser Therapies, Low-Level)) OR (Laser Therapy, Low Level)) OR (Low-Level Laser Therapies)) OR (Laser Irradiation, Low-Power)) OR (Irradiation, Low-Power Laser)) OR (Laser Irradiation, Low Power)) OR (Low-Power Laser Therapy)) OR (Low Power Laser Therapy)) OR (Laser Therapy, Low-Power)) OR (Laser Therapies, Low-Power)) OR (Laser Therapy, Low Power)) OR (Low-Power Laser Therapies)) OR (Low-Level Laser Therapy)) OR (Low Level Laser Therapy)) OR (Low-Power Laser Irradiation)) OR (Low Power Laser Irradiation)) OR (Laser Biostimulation)) OR (Biostimulation, Laser)) OR (Laser Phototherapy)) OR (Phototherapy, Laser)) OR (Phototherapy[MeSH Terms])) OR (Phototherapies)) OR (Therapy, Photoradiation)) OR (Photoradiation Therapies)) OR (Therapies, Photoradiation)) OR (Light Therapy)) OR (Light Therapies)) OR (Therapies, Light)) OR (Therapy, Light)) OR (Photoradiation Therapy)
2. (blue light) OR (visible light)
3. (((wound healing[MeSH Terms]) OR (Healing, Wound)) OR (Healings, Wound)) OR (Wound Healings)
4. Skin
5. (((#1) AND (#2)) AND (#3)) AND (#4)

Web of Science Search Formula

1. (((((ALL=(Low-Level Light Therapy)) OR ALL=(Photobiomodulation Therapy)) OR ALL=(LLLT)) OR ALL=(Laser Therapy)) OR ALL=(Phototherapy)) OR ALL=(Laser Biostimulation)
2. ((ALL=(BLUE LIGHT)) OR ALL=(VISIBLE LIGHT)) OR ALL=(visible light phototherapy)
3. ALL=(WOUND HEALING)
4. ALL=(SKIN)
5. #4 AND #3 AND #2 AND #1
6. #4 AND #3 AND #2 AND #1 and 2022 or 2021 or 2020 or 2019 or 2018 or 2017 or 2016 or 2015 or 2014 or 2013 or 2012

SCOPUS Search Formula

1. (TITLE-ABS-KEY (*low-level* AND *light* AND *therapy*) OR TITLE-ABS-KEY (*photobiomodulation* AND *therapy*) OR TITLE-ABS-KEY (*photobiomodulation*) OR TITLE-ABS-KEY (*laser* AND *therapy*) OR TITLE-ABS-KEY (*phototherapy*) OR TITLE-ABS-KEY (*laser* AND *biostimulation*))
2. (TITLE-ABS-KEY (*blue* AND *light*) OR TITLE-ABS-KEY (*visible* AND *light*))
3. TITLE-ABS-KEY (*wound* AND *healing*)
4. TITLE-ABS-KEY (*skin*)

CINHAL Search Formula

1. low-level light therapy OR photobiomodulation therapy OR lllt OR Laser Therapy OR Phototherapy OR Laser Biostimulation
2. visible light OR blue light
3. wound healing
4. skin
5. (skin) AND (S1 AND S2 AND S3 AND S4)
